# Supplementary material for: Abundance estimation for line transect sampling: A comparison of distance sampling and spatial capture-recapture models
Source: PLoS One. 2021 May 28;16(5):e0252231. doi: 10.1371/journal.pone.0252231 (PMC8162584; doi:10.1371/journal.pone.0252231)
Supplement: S1 Appendix — (DOCX) [file pone.0252231.s001.docx]

**S1 Appendix. *Supplementary results***

We present estimates for additional parameters, g(0), σ_d_, σ_m_, β_0_, and β_1_ estimated from scenarios listed in the manuscripts main body (Table S1.1).

We evaluated additional modifications to several scenarios included in the main body of the manuscript. Specifically, we doubled the number of occasions for the g(0) = 1 single-observer scenario from four to eight and the g(0) = 0.8 double-observer scenario from 5 to 10. Additionally, for all double-observer scenarios, we compared analyses of reduced spatial capture-recapture (SCR) datasets, where detections were recorded and modeled as binary, i.e. detected by at least one observer, or not detected, to full datasets where detections were recorded and analyzed normally, i.e. detected by two observers, detected by one observer, or not detected. We simulated and analyzed 1000 datasets for each of these scenarios. Finally, we analyzed datasets simulated under two variations of the spatial covariate scenario described in the main body of the manuscript using the uniform SCR and DS models to test their sensitivity to misspecification. We simulated 100 datasets under each spatial covariate scenario. In one scenario density followed the description from the main body of the manuscript, i.e., density was a linear function of the spatial covariate. In the other scenario, density was a quadratic function of the spatial covariate with density reaching its maximum at intermediate covariate values, in the middle of the state space where survey effort was highest.

Bias and root mean squared error of abundance estimates decreased for both distance sampling and spatial capture-recapture models in the single- and double-observer scenarios where the number of occasions was doubled (Table S1.2). Abundance credible interval width decreased ~25% and ~50% for SCR and DS models, respectively, in comparison to scenarios described in the body of the manuscript (Table S1.2). Abundance credible interval coverage decreased for DS, becoming even less nominal, but remained nominal for SCR (Table S1.2).

In the comparison of reduced to full double-observer datasets, SCR abundance estimates of the reduced datasets increased root mean squared error between 7% and 25% (Table S1.3). This increase in error was largest when g(0) was 0.8 and lowest when g(0) was 0.3. The reduced dataset increased bias of abundance estimates only when g(0) was 0.8 (Table S1.3). Estimates were similarly biased when g(0) was 0.5 and 0.3. Abundance credible intervals for the reduced datasets were between 4% and 10% wider than those for the full datasets, with the increase in width being greatest when g(0) was 0.8 and smallest when g(0) was 0.3 (Table S1.3).

We present the average posterior median for abundance and its RMSE and bias for all scenarios listed in the main body of this paper (Table S1.4). The median generally estimated abundance with lower error and bias than did the posterior mean.

We present the average posterior mean for abundance, its average credible interval, RMSE, and bias for mispecified DS and SCR models, where datasets were simulated such that density was a function of a spatial covariate and were analyzed with a model that assumed density was constant across space (Table S1.5). SCR had lower bias and RMSE than DS in both scenarios, although both models were substantially positively biased in the scenario where density was a quadratic function of the spatial covariate.

Table S1.1. Model performance based on simulated datasets for spatial capture-recapture models (SCR) and distance sampling models (DS), where g(0) (detection on the transect line) was either fixed at the true value, informed via a prior distribution, or estimated via a double observer sampling protocol (i.e. mark-recapture distance sampling). The average posterior mean and 95% credible interval (CI) were calculated for g(0), detection scale (σ_d_), movement scale (σ_m_), and parameters of the spatial density model (β_0_ and β_1_) across simulations. Additionally, the 95% CI coverage, root mean squared error (RMSE) and bias of the posterior mean were calculated for each of these parameters.

| **g(0)** | **# Occasions** | **# Observers** | **Model** | **Parameter** | **Mean** | **95% CI** | **95% CI Coverage** | **RMSE** | **Bias** |
| --- | --- | --- | --- | --- | --- | --- | --- | --- | --- |
| 1 | 4 | 1 | SCR | σ_d_ | 0.34 | (0.28, 0.41) | 0.946 | 0.035 | 0.005 |
|  |  |  |  | 1/(2σ_m_^2^) | 0.19 | (0.10, 0.30) | 0.929 | 0.07 | 0.027 |
|  |  |  | DS–fixed g(0) | σ_d_ | 0.35 | (0.28, 0.46) | 0.941 | 0.053 | 0.014 |
| 0.8 | 6 | 1 | SCR | g(0) | 0.74 | (0.50, 0.96) | 0.968 | 0.122 | -0.061 |
|  |  |  |  | σ_d_ | 0.35 | (0.29, 0.43) | 0.952 | 0.039 | 0.014 |
|  |  |  |  | 1/(2σ_m_^2^) | 0.18 | (0.11, 0.28) | 0.926 | 0.055 | 0.024 |
|  |  |  | DS–fixed g(0) | σ_d_ | 0.35 | (0.28, 0.44) | 0.943 | 0.045 | 0.012 |
|  |  |  | DS–informed g(0) | g(0) | 0.79 | (0.55, 0.95) | 1 | 0.014 | -0.014 |
|  |  |  |  | σ_d_ | 0.35 | (0.28, 0.44) | 0.942 | 0.046 | 0.012 |
| 0.8 | 5 | 2 | SCR | g(0) | 0.77 | (0.62, 0.90) | 0.945 | 0.079 | -0.027 |
|  |  |  |  | σ_d_ | 0.34 | (0.29, 0.40) | 0.958 | 0.027 | 0.008 |
|  |  |  |  | 1/(2σ_m_^2^) | 0.17 | (0.11, 0.25) | 0.95 | 0.04 | 0.013 |
|  |  |  | DS–double observer | g(0) | 0.77 | (0.61, 0.90) | 0.938 | 0.084 | -0.032 |
|  |  |  |  | σ_d_ | 0.34 | (0.29, 0.42) | 0.949 | 0.032 | 0.011 |
| 0.5 | 10 | 1 | SCR | g(0) | 0.51 | (0.33, 0.74) | 0.953 | 0.106 | 0.013 |
|  |  |  |  | σ_d_ | 0.34 | (0.28, 0.42) | 0.945 | 0.036 | 0.006 |
|  |  |  |  | 1/(2σ_m_^2^) | 0.18 | (0.11, 0.26) | 0.933 | 0.048 | 0.016 |
|  |  |  | DS–fixed g(0) | σ_d_ | 0.34 | (0.28, 0.43) | 0.942 | 0.04 | 0.009 |
|  |  |  | DS–informed g(0) | g(0) | 0.48 | (0.28, 0.68) | 1 | 0.022 | -0.022 |
|  |  |  |  | σ_d_ | 0.34 | (0.28, 0.43) | 0.941 | 0.04 | 0.009 |
| 0.5 | 7 | 2 | SCR | g(0) | 0.49 | (0.36, 0.65) | 0.953 | 0.076 | -0.006 |
|  |  |  |  | σ_d_ | 0.34 | (0.29, 0.41) | 0.946 | 0.033 | 0.007 |
|  |  |  |  | 1/(2σ_m_^2^) | 0.18 | (0.11, 0.25) | 0.944 | 0.042 | 0.015 |
|  |  |  | DS–double observer | g(0) | 0.48 | (0.30, 0.66) | 0.934 | 0.097 | -0.024 |
|  |  |  |  | σ_d_ | 0.34 | (0.29, 0.42) | 0.939 | 0.037 | 0.01 |
| 0.3 | 20 | 1 | SCR | g(0) | 0.31 | (0.20, 0.44) | 0.95 | 0.059 | 0.006 |
|  |  |  |  | σ_d_ | 0.34 | (0.28, 0.41) | 0.951 | 0.033 | 0.007 |
|  |  |  |  | 1/(2σ_m_^2^) | 0.17 | (0.11, 0.24) | 0.922 | 0.04 | 0.012 |
|  |  |  | DS–fixed g(0) | σ_d_ | 0.34 | (0.29, 0.42) | 0.944 | 0.037 | 0.01 |
|  |  |  | DS–informed g(0) | g(0) | 0.27 | (0.11, 0.48) | 1 | 0.036 | -0.033 |
|  |  |  |  | σ_d_ | 0.34 | (0.29, 0.42) | 0.942 | 0.036 | 0.01 |
| 0.3 | 11 | 2 | SCR | g(0) | 0.31 | (0.21, 0.43) | 0.96 | 0.054 | 0.009 |
|  |  |  |  | σ_d_ | 0.34 | (0.29, 0.41) | 0.94 | 0.033 | 0.006 |
|  |  |  |  | 1/(2σ_m_^2^) | 0.18 | (0.11, 0.25) | 0.931 | 0.042 | 0.015 |
|  |  |  | DS–double observer | g(0) | 0.29 | (0.15, 0.47) | 0.947 | 0.083 | -0.007 |
|  |  |  |  | σ_d_ | 0.34 | (0.29, 0.42) | 0.932 | 0.037 | 0.009 |
| 0.8 | 10 | 1 | SCR–spatial covariate | β_0_ | -4.49 | (-5.35, -3.69) | 0.929 | 0.345 | -0.035 |
|  |  |  |  | β_1_ | 0.9 | (0.59, 1.24) | 0.934 | 0.137 | 0.009 |
|  |  |  |  | g(0) | 0.78 | (0.60, 0.78) | 0.985 | 0.066 | -0.018 |
|  |  |  |  | σ_d_ | 0.68 | (0.58, 0.96) | 0.955 | 0.034 | 0.015 |
|  |  |  |  | 1/(2σ_m_^2^) | 0.03 | (0.02, 0.04) | 0.96 | 0.004 | 0.001 |
|  |  |  | DS–fixed g(0)– spatial covariate | β_0_ | -4.65 | (-5.37, -3.95) | 0.775 | 0.486 | -0.193 |
|  |  |  |  | β_1_ | 0.95 | (0.66, 1.24) | 0.794 | 0.186 | 0.058 |
|  |  |  |  | σ_d_ | 0.69 | (0.61, 0.80) | 0.919 | 0.041 | 0.023 |
|  |  |  | DS–informed g(0)–spatial covariate | β_0_ | -4.6 | (-5.34, -3.89) | 0.789 | 0.469 | -0.144 |
|  |  |  |  | β_1_ | 0.93 | (0.64, 1.23) | 0.796 | 0.181 | 0.039 |
|  |  |  |  | g(0) | 0.8 | (0.69, 0.89) | 1 | 0.001 | 0 |
|  |  |  |  | σ_d_ | 0.68 | (0.61, 0.80) | 0.914 | 0.041 | 0.23 |

Table S1.2. Abundance estimate properties for spatial capture-recapture (SCR) and distance sampling (DS) models.

| Model | Number of Observers | Number of Occasions | g(0) | Mean | Median | 95% CI | 95% CI Coverage | Mean-RMSE | Median-RMSE | Posterior-RMSE | Mean-Bias | Median-Bias |
| --- | --- | --- | --- | --- | --- | --- | --- | --- | --- | --- | --- | --- |
| SCR | 1 | 4 | 1 | 104.330 | 103.042 | (72.99, 143.05) | 0.944 | 343.086 | 334.640 | 668.244 | 4.330 | 3.042 |
| SCR | 1 | 8 | 1 | 101.563 | 100.705 | (76.67, 131.34) | 0.956 | 193.924 | 192.219 | 391.020 | 1.563 | 0.705 |
| DS | 1 | 4 | 1 | 102.207 | 100.932 | (72.79, 138.90) | 0.854 | 509.714 | 502.034 | 802.519 | 2.207 | 0.932 |
| DS | 1 | 8 | 1 | 100.125 | 99.486 | (79.06, 124.82) | 0.803 | 325.732 | 325.061 | 464.415 | 0.125 | -0.514 |
| SCR | 2 | 5 | 0.8 | 103.579 | 102.551 | (76.145, 136.825) | 0.956 | 244.455 | 238.291 | 487.312 | 3.579 | 2.551 |
| SCR | 2 | 10 | 0.8 | 102.327 | 101.599 | (79.406, 129.362) | 0.96 | 150.059 | 147.353 | 314.175 | 2.327 | 1.599 |
| DS | 2 | 5 | 0.8 | 103.874 | 102.716 | (80.319, 134.077) | 0.819 | 389.065 | 378.762 | 584.275 | 3.873 | 2.716 |
| DS | 2 | 10 | 0.8 | 102.221 | 101.701 | (85.641, 121.755) | 0.725 | 285.417 | 283.356 | 371.725 | 2.221 | 1.701 |

Table S1.3. Abundance estimate properties for spatial capture-recapture analyses of full and reduced double-observer datasets. Reduced datasets treated detection events as either detected by at least one observer or not detected, whereas full datasets treated detection events as detected by two observers, detected by one observer, or not detected.

| Dataset | g(0) | Mean | Median | 95% CI | 95% CI Coverage | Mean-RMSE | Median-RMSE | Posterior-RMSE | Mean-Bias | Median-Bias |
| --- | --- | --- | --- | --- | --- | --- | --- | --- | --- | --- |
| Reduced | 0.8 | 106.402 | 105.128 | (77.066, 143.210) | 0.945 | 305.330 | 289.320 | 596.466 | 6.402 | 5.128 |
| Full | 0.8 | 103.579 | 102.551 | (76.145, 136.825) | 0.956 | 244.455 | 238.291 | 487.312 | 3.579 | 2.551 |
| Reduced | 0.5 | 104.830 | 103.390 | (75.016, 142.854) | 0.96 | 289.038 | 276.208 | 596.235 | 4.830 | 3.390 |
| Full | 0.5 | 104.963 | 103.753 | (76.209, 140.545) | 0.952 | 263.002 | 252.321 | 536.993 | 4.963 | 3.753 |
| Reduced | 0.3 | 104.652 | 103.234 | (74.619, 142.905) | 0.947 | 314.247 | 301.454 | 625.299 | 4.652 | 3.234 |
| Full | 0.3 | 104.666 | 103.379 | (75.500, 141.131) | 0.948 | 291.900 | 280.399 | 578.570 | 4.666 | 3.379 |

Table S1.4. Model performance based on simulated datasets for spatial capture-recapture models (SCR) and distance sampling models (DS), where g(0) (detection on the transect line) was either fixed at the true value, informed via a prior distribution, or estimated via a double observer sampling protocol (i.e. mark-recapture distance sampling). The average posterior median and the bias and root mean squared error (RMSE) were calculated for abundance.

| **g(0)** | **# Occasions** | **# Observers** | **Model** | **Median** | **RMSE-Median** | **Bias-Median** |
| --- | --- | --- | --- | --- | --- | --- |
| 1 | 4 | 1 | SCR | 103.0 | 18.3 | 3.0 |
|  |  |  | DS–fixed g(0) | 100.9 | 22.4 | 0.9 |
| 0.8 | 6 | 1 | SCR | 107.1 | 20.2 | 7.1 |
|  |  |  | DS–fixed g(0) | 100.0 | 21.9 | 0.0 |
|  |  |  | DS–informed g(0) | 101.4 | 22.2 | 1.4 |
| 0.8 | 5 | 2 | SCR | 102.6 | 15.4 | 2.6 |
|  |  |  | DS–double observer | 102.7 | 19.5 | 2.7 |
| 0.5 | 10 | 1 | SCR | 103.6 | 18.7 | 3.6 |
|  |  |  | DS–fixed g(0) | 101.6 | 21.1 | 1.6 |
|  |  |  | DS–informed g(0) | 107.1 | 23.3 | 7.1 |
| 0.5 | 7 | 2 | SCR | 103.8 | 15.9 | 3.8 |
|  |  |  | DS–double observer | 108.8 | 27.7 | 8.8 |
| 0.3 | 20 | 1 | SCR | 102.4 | 17.3 | 2.4 |
|  |  |  | DS–fixed g(0) | 99.7 | 20.8 | -0.3 |
|  |  |  | DS–informed g(0) | 117.1 | 30.4 | 17.1 |
| 0.3 | 11 | 2 | SCR | 103.4 | 16.7 | 3.4 |
|  |  |  | DS–double observer | 115.9 | 46.5 | 15.9 |
| 0.8 | 10 | 1 | SCR–spatial covariate | 153.6 | 17.7 | 3.6 |
|  |  |  | DS–fixed g(0)– spatial covariate | 151.9 | 20.0 | 1.9 |
|  |  |  | DS–informed g(0)–spatial covariate | 150.8 | 19.2 | 0.8 |

Table S1.5. Abundance estimate properties for distance sampling and spatial capture-recapture analyses of datasets simulated such that density was a function of spatial covariates for a population of 150 individuals. Density was a linear function of covariates in the linear simulation models, such that $density=\exp\left( -4.459+0.895*Covariate \right)$. Density was a quadratic function of covariates in the quadratic simulation models, such that $density=\exp\left( -{Covariate}^{2}+3.6*Covariate-4.789 \right)$. Covariate values ranged from 0 to 3.6. Simulated datasets were analyzed with spatial capture-recapture and distance sampling models that modeled density as constant across space. The distance sampling model fixed g(0) at the true simulating value of 0.8.

| **g(0)** | **# of Occasions** | **# of Observers** | **Model** | **Mean** | **95% CI** | **Mean-RMSE** | **Mean-Bias** |
| --- | --- | --- | --- | --- | --- | --- | --- |
| 0.8 | 10 | 1 | SCR - linear | 148.8 | (116.0, 189.5) | 24.97399 | -1.2 |
|  |  |  | DS - linear | 158.3 | (127.4, 193.8) | 31.67333 | 8.3 |
|  |  |  | SCR - quadratic | 221.7 | (185.6, 262.8) | 76.28761 | 71.7 |
|  |  |  | DS - quadratic | 331.9 | (284.6, 383.8) | 187.0866 | 181.9 |
