# Supplementary material for: Abundance estimation for line transect sampling: A comparison of distance sampling and spatial capture-recapture models
Source: PLoS One. 2021 May 28;16(5):e0252231. doi: 10.1371/journal.pone.0252231 (PMC8162584; doi:10.1371/journal.pone.0252231)
Supplement: S2 Appendix — (DOCX) [file pone.0252231.s002.docx]

**S2 Appendix. *Effects of movement on distance sampling abundance estimation***

We evaluated the precision of conventional distance sampling abundance estimates under a range of individual movement scenarios. We simulated spatial capture-recapture datasets following the description in the Material & Methods section with all combinations of the movement scale parameter, σ_m_, ranging from 0.5 to 8 at increments of 0.5, and 4, 8, or 12 sampling occasions. However, we discarded all detections that occurred outside the endpoints of line transects. For each combination we simulated 1000 datasets across a 70 by 70 unit state space. The state space was large enough for an individual with an activity center at the edge of the state space to be at least three σ_m_ away from the closest point on a transect line for the largest evaluated σ_m_. This resulted in uniformly distributed density in the sampled region for all values of σ_m_. We simulated a population of 400 individuals with the same line transect configuration as in the Methods section, with g(0) = 1 and σ_d_ = 1/3. We used the Distance package (Miller et al., 2019) in program R to estimate abundance and its confidence interval for each simulation. We aggregated confidence interval coverage across each combination of the number of occasions an σ_m_.

Confidence interval coverage varied across both σ_m_ and the number of occasions (Figure S2.1). When σ_m_ was 0.5, coverage was nominal for all three occasion scenarios, but coverage dropped to below nominal for all other values of σ_m_. Coverage was smallest between σ_m_= 2 and σ_m_= 3 for the three sets of occasions, and slowly increased as σ_m_ increased above three. Additionally, coverage decreased as the number of occasions increased across most values of σ_m_.

In these simulations, transect lines were parallel to one another and placed two units apart. We suspect that the below nominal confidence interval coverage is due to spatial autocorrelation in the number of detections on nearby transect lines caused by the scale of individuals’ movements relative to the spacing of the transect lines. Indeed, over-precision peaks when the scale of individuals’ movements is similar to the spacing of transect lines and increases with the number of sampling occasions (Figure S2.1). As the movement scale decreases from this peak, confidence interval coverage becomes closer to nominal, as individuals are more likely to be seen along the same transect line across occasions than by different, adjacent lines. As the movement scale increases from this peak, confidence interval coverage again becomes closer to nominal, as individuals may be seen across a larger set of lines.

*Bootstrapping confidence intervals*

Variance and confidence intervals of abundance are more robust when generated via bootstrapping over transect lines with replacement than when derived analytically (Thomas et al. 2002). Therefore, we tested whether bootstrapping would fix or improve the over-precision issue described above. We simulated and analyzed an additional 100 datasets following the same specifications as described above with σ_m_ = 2.5 and 10 sampling occasions. We bootstrapped confidence intervals for each simulated dataset whose abundance confidence interval, derived via the Distance package, did not contain the true value of abundance. For each of these datasets we bootstrapped over transect lines with replacement 1000 times, estimated abundance for each bootstrapped dataset, and derived 95% confidence intervals from the 2.5 and 97.5 percentiles of the bootstrapped abundance estimates.

Of the 100 confidence intervals generated by the Distance package, 21 did not contain the true value of abundance. The bootstrapped confidence intervals did not contain the true value of abundance in 19 of the 21 datasets analyzed. Assuming that the bootstrapped confidence intervals for all other datasets would include the true value, this would still result in an estimated 95% confidence interval coverage of 81%, which is well below nominal (p < 0.0001 according to the binomial test). Other bootstrapping approaches including a parametric bootstrap (Hedley and Buckland 2004) and moving block bootstrap (Efron and Tibishirani 1993) may provide nominal coverage but were not tested here.


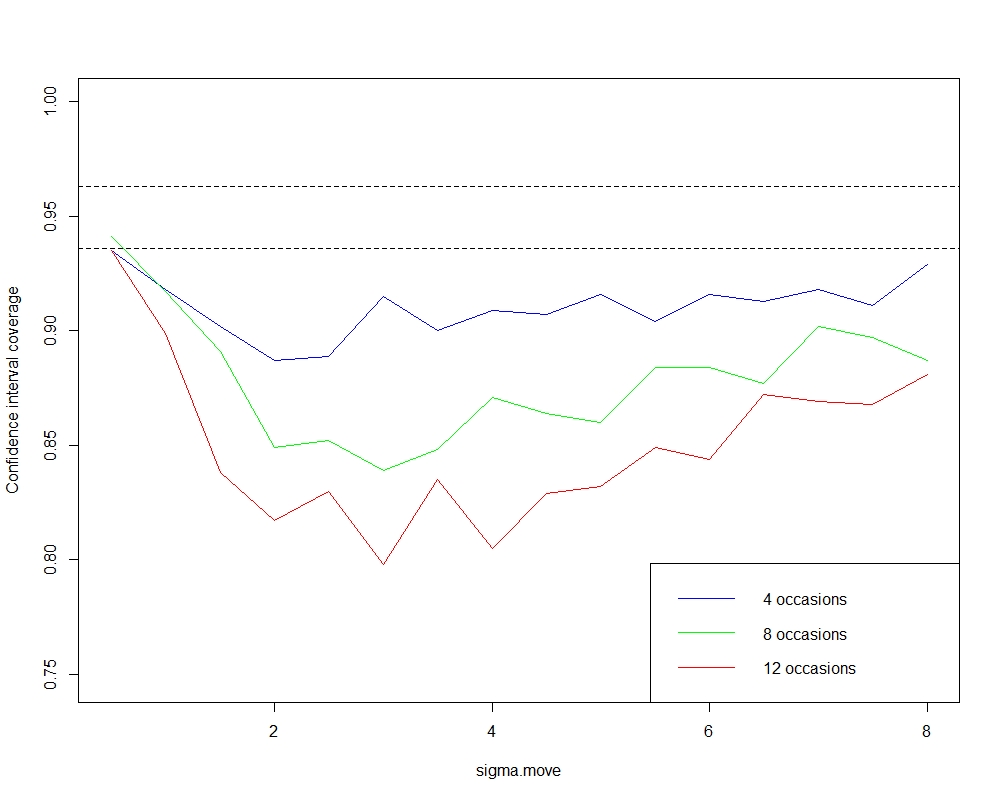


Figure S2.1. Confidence interval coverage of abundance estimated using distance sampling. There were 1000 datasets simulated and analyzed for each combination of the movement scale, x-axis, and the number of occasions, line color. The dashed horizontal lines bound nominal confidence interval coverage.

**References**

Efron, B. and Tibshirani, R. J. 1993. An introduction to the bootstrap. – Chapman Hall/CRC. FL, USA.

Hedley, S. L. and Buckland, S. T. 2004. Spatial models for line transect sampling. – J. Agr. Biol. Envir. St. 9: 181–199.

Miller D. L. et al. 2019. Distance sampling in R. *–* J. Stat. Softw. 89: 1–28.

Thomas, L. et al. 2002. Distance sampling. – Encyclopedia of Environmetrics 1: 544–552
